# Progress Testing in UK Medical Education: Evaluating Its Impact and Potential

**DOI:** 10.7759/cureus.52607

**Published:** 2024-01-20

**Authors:** Ghulam M Majeed, Juned Islam, Girinath Nandakumar, KarYen Phoong

**Affiliations:** 1 Faculty of Health, Education, Medicine, and Social Care, Anglia Ruskin University, Cambridge, GBR; 2 Department of Psychiatry, Whittington Hospital, London, GBR; 3 Department of Trauma and Orthopaedics, East Lancashire Healthcare Trust, Blackburn, GBR; 4 Department of General Practice, East Lancashire Healthcare Trust, Blackburn, GBR

**Keywords:** ukmla, quality assurance in education, assessment methods, uk medical licensing assessment, uk medical curriculum, progress testing, medical education

## Abstract

This comprehensive review critically examines the UK medical curriculum, with a particular focus on progress testing as an innovative assessment strategy. The curriculum, evolving from foundational sciences to practical applications, is encapsulated in the integrated curriculum model (ICM). This model adeptly combines theoretical knowledge with clinical practice, fostering cognitive, affective, and psychomotor skills among medical students.

Central to this review is an exploration of progress testing. This method, grounded in constructivist learning theories, emphasises continuous assessment and professional development. Progress testing's regular, comprehensive examinations are instrumental in guiding students through the progressive stages of competence, as outlined in Miller's pyramid, from foundational knowledge to clinical proficiency.

The review also addresses the broader impacts of progress testing on teaching approaches, student feedback, academic and pastoral support, and quality assurance. By aligning with the dynamic requirements of 21st-century medical training, progress testing not only nurtures well-rounded professionals but also ensures compliance with regulatory bodies like the General Medical Council. Its emphasis on continuous evaluation aligns with the practical realities of a medical career, driving curricular innovation and aligning with regulatory standards.

The implementation of progress testing marks a significant advancement in medical education. Its continuous, holistic nature benefits both students and educators, nurturing a more engaged learning attitude and meeting evolving medical needs. The adoption of this assessment strategy is seen as pivotal in shaping competent medical professionals, ready to face the challenges of modern medical practice.

## Introduction and background

The UK medical curriculum stands as a testament to the comprehensive and evolving nature of medical education [1]. Rooted in a deep understanding of core medical sciences and expanding into practical, real-world applications, this curriculum is designed to equip students to become competent and empathetic doctors. The integration of various teaching methodologies, including the innovative integrated curriculum model (ICM), ensures that students are not only well-versed in theory but also adept at applying their knowledge in diverse clinical settings. However, this sophisticated system faces its challenges. One key issue is balancing broad educational goals with the pressures of exam-oriented learning, a dilemma that often affects student focus and well-being. As a response to these challenges, progress testing emerges as a progressive and holistic assessment strategy, deeply rooted in constructivist learning theories [2]. This method promises a more balanced and continuous evaluation of medical knowledge and skills, aligning closely with the practical realities of a medical career. This review delves into the structure, strengths, and challenges of the UK medical curriculum, critically analysing the role of progress testing in shaping the future of medical education.

## Review

Structure and background of the UK medical curriculum

The medical degree curriculum in the UK, spanning four to six years, is strategically designed to equip students with the necessary skills and expertise to become competent doctors [3,4]. The program typically lays a foundation in the early years, through guided lectures, problem-based tutorials, demonstrations, coursework, and practical workshops. These focus on core medical sciences such as anatomy, physiology, and biochemistry, aptly named the pre-clinical years [5]. As students progress, the curriculum evolves to build upon these foundations, incorporating more tailored learning methods. These include simulated scenarios, opportunities for research, quality improvement projects, portfolio development, and self-designed elective placements, fostering student confidence, maturity, and autonomy. The ICM, as outlined by Brauer and Ferguson, builds upon and contextualises the knowledge base acquired in the pre-clinical years into the later stages of medical training, ensuring a cohesive and progressive educational journey for medical students [6].

As the curriculum advances from foundational knowledge to more complex clinical skills, the assessment methods also evolve, reflecting a shift from theoretical understanding to practical application and proficiency. Throughout the ICM, students are subject to a variety of formative and summative assessments [7]. These include written examinations, objective structured clinical examinations (OSCEs), portfolio submissions, extra-curricular modules, student-selected components (SSC), and the Prescribing Safety Assessment (PSA). Moreover, most medical schools encourage students to undertake an additional degree, usually an intercalated Bachelor of Science (BSc). This is an opportunity for medical students to take a year out of their medical degree to delve deeper into a specific area of interest, enhancing their knowledge base and research experience [8]. This multimodal approach addresses various educational preferences and needs, aligning with Fleming's visual, aural, read/write, and kinesthetic (VARK) model for diverse student engagement [9]. This ensures that medical students are thoroughly tested on the plethora of skills essential for their future careers.

Strengths and challenges of the ICM

The strengths of the ICM lie in its blend of teaching and assessment methods, which caters to a variety of learning styles. It offers a robust educational experience that develops students' cognitive skills through problem-solving and critical thinking, affective skills by promoting empathy and ethical understanding, and psychomotor skills through hands-on clinical practice, for instance, Bleakley's 'apprenticeship' model, which integrates practical ward-based learning for final-year medical students, blending theoretical knowledge with hands-on clinical experience. This 'apprenticeship' approach facilitates a seamless transition into the medical profession, complementing the diverse learning strategies of the ICM [10].

Despite these strengths, the ICM faces certain challenges that warrant attention. One notable concern, highlighted by Lovell, is the curriculum's vulnerability to 'coaching', where the primary attention of students may shift from the holistic objective of becoming a competent doctor to narrowly concentrating on passing exams for career advancement and portfolio building [11]. This shift can dilute the depth of learning and impede professional development. Furthermore, the stress induced by frequent examinations can negatively impact students' mental health and learning efficacy [12]. Some critiques, such as those by Sbayeh et al., suggest that while assessing many competencies are crucial, not all are directly pertinent to the routine responsibilities of a foundation doctor. For example, extensive anatomy knowledge may not always correlate with the applied basics of foundation doctors [13]. This mismatch indicates a need for curriculum and exam format revisions, emphasising content more relevant to foundation doctors and reducing focus on specialised topics with limited practical application.

Arnold and Willoughby advocate for progress testing, a form of assessment tailored to medical education [2]. This method involves systematic, curriculum-spanning examinations that focus on assessing cumulative knowledge. It is designed to reflect the continuous learning essential in clinical practice, thus aligning more effectively with the professional requirements of future doctors. This approach represents a shift from traditional testing methods, emphasising the relevance and applicability of assessments in forming competent medical practitioners.

Underlying philosophy of progress testing

Progress testing is an assessment method rooted in constructivist learning theories. Originating from Piaget's theory on cognitive development, constructivism emphasises the retention of knowledge and its everyday application in clinical settings [14]. This approach is well-reflected in progress testing, where students are continually encouraged to connect new medical information with their prior understanding, thereby enhancing their comprehension and critical thinking skills.

Constructivist theories, particularly Vygotsky's social constructivism, emphasise the necessity of context and social dynamics in the learning process [15]. Progress testing integrates the sociocultural elements of Vygotsky's work by presenting scenarios that mimic real-life clinical situations. This method fosters students' ability to apply their learning in contextually relevant and socially interactive settings. Additionally, it ensures that progress testing examines not only students' knowledge but also their ability to effectively use that knowledge in diverse and dynamic environments.

Progress testing cumulatively evaluates medical students against all objectives of their curriculum [16]. This method involves frequently presenting a comprehensive set of questions covering the full scope of their studies, rather than focusing only on recent or specific topics. Such a strategy consistently prompts students to build upon and synthesise their knowledge. This leads to a deeper, more cohesive grasp of medical concepts. It not only solidifies theoretical understanding but also boosts practical application, equipping students for the complex challenges of their medical careers [16].

Evaluating the effectiveness of progress testing is crucial, and Miller's pyramid of competence is a key reference in this regard. This framework categorises the stages of professional development into four levels: 'knows', 'knows how', 'shows how', and 'does' [17]. Progress testing is uniquely designed to guide medical students through these successive stages. It begins with assessing and reinforcing foundational knowledge ('knows') and then progresses to evaluating their ability to apply this knowledge in theoretical scenarios ('knows how'). As they advance in their studies, progress testing challenges students to demonstrate their skills in simulated environments ('shows how'). Ultimately, in the most advanced stages, students are expected to competently perform these skills in clinical settings ('does'). This systematic approach, deeply aligned with constructivist learning theories, ensures not just the acquisition of knowledge but also its applied application. Through this, progress testing significantly contributes to the progression of holistic, proficient physicians, adept at both understanding medical concepts and applying them effectively in their clinical practice.

Curriculum roadmapping and outcomes

Progress testing effectively consolidates the fragmented, departmentalised approach to medical education, as described by Harden [18]. This emphasis on a curriculum with specific objectives provides students with a clear roadmap of the expectations upon graduation and staff with a clearly definition of their roles within the curriculum [18]. Administered multiple times throughout the year, typically three to four times, with each test comprising of 100-180 questions, progress tests continuously challenge students with a broad spectrum of content [19]. This thorough and repetitive exposure ensures an comprehensive coverage and understanding of the entire curriculum, effectively preparing students for the varied demands of their future clinical roles.

This approach represents a fundamental shift in focus from simply preparing for the next exam to broader preparation for a career in medicine. By testing students repeatedly across the entire curriculum, progress testing facilitates spaced repetition, a crucial factor for junior doctors needing to recall and apply a vast array of information in diverse settings [20]. Techniques like cumulative assessments and repeated exposure to core concepts are instrumental in solidifying this knowledge, making it more accessible in practical scenarios.

Professional and regulatory statutory body requirements

The General Medical Council (GMC) plays a pivotal role in assuring the quality of medical programs across the UK. As the regulatory body for doctors in the UK, it sets detailed standards for medical education and training, encompassing curriculum content, teaching methodologies, student assessment, and the overall competence of graduates. These standards are essential, as all medical schools in the UK must comply with them to obtain and maintain accreditation [3,4]. 

Progress testing, aligned with the GMC's Good Medical Practice, emphasises continuous assessment and professional development. It evaluates students against their curriculum's goals from the outset of their education, ensuring comprehensive examination of their knowledge, skills, and behaviour. This approach meets the GMC's criteria, aiding in the growth of competent professionals and helping medical schools meet rigorous accreditation standards [21]. Furthermore, progress testing resonates with the GMC's focus on student motivation and engagement, by setting and regularly addressing clear, long-term objectives. This helps students appreciate and commit to their studies, supporting its integration into medical curricula. However, its adoption requires careful consideration of GMC compliance and adjustments to existing educational structures.

Teaching approach

The teaching approach centred around progress testing transforms the educational landscape significantly. It moves beyond mere exam-centric studying, instead preparing students for tangible scenarios that they will encounter throughout their careers. This approach offers a curriculum better aligned with real-world medical scenarios, thereby making the learning experience more relevant and applicable.

Beyond shaping the curriculum, progress testing also addresses key educational challenges. One of these key benefits is its ability to mitigate the drawbacks of rote learning, a common issue in traditional examination methods which assess recently covered specific sections of the curriculum [22]. These exams are often split into specific domains such as physiology, anatomy, and pharmacology. Progress testing draws on these multiple domains of medicine; thus, students are encouraged to engage deeply with the material, fostering better knowledge retention, rather than just memorising facts as demanded by traditional exams. This deeper engagement not only improves understanding but also increases student motivation. Students tend to realise the importance of truly comprehending the subject matter due to the ongoing nature of progress testing.

Furthermore, progress testing introduces a higher degree of flexibility in teaching techniques. It opens up possibilities for incorporating diverse teaching styles, like case studies, interactive lectures, and Barrows' problem-based learning, where students learn from analysing, researching, and solving real-world scenarios [23]. This adaptability caters to different learning preferences, creating a more inclusive and stimulating environment. Such flexibility enables educators to tailor their teaching strategies to complement the continuous and comprehensive nature of progress testing. As a result, teaching becomes not just more effective but also dynamic, adapting to meet the evolving needs of students.

Anonymity and feedback

Progress testing stands out as an unbiased objective form of assessment, primarily due to its administration as a completely anonymous exam. This anonymity is key in minimising implicit biases, ensuring that students are evaluated solely on their responses [24]. Additionally, the standardised format of questions and the extensive coverage of content across the curriculum contribute to the objectivity of this examination method. This structure facilitates the generation of quantifiable data that accurately reflects students' understanding and skills.

The data generated from progress testing is invaluable for providing detailed feedback to students. Feedback can be conveyed through various channels, including personalised reports, digital platforms, or individual meetings with educators. Typically, this feedback provides insights into areas where students excel or need improvement, tracks their progress over time, and enables comparison with peer performance. Such bespoke feedback is crucial in guiding students' future learning strategies, helping them focus on areas for growth and reinforcing their strengths [25]. As highlighted by Wass et al., this form of feedback is essential for students to identify specific sections of the curriculum that may require added attention, offering a clear view of their academic journey and performance relative to established benchmarks [25].

Moreover, this personalised feedback significantly enhances self-directed learning [26]. Receiving clear, actionable insights into their performance empowers students to take control of their education. They can identify specific topics requiring more attention, adjust their study methods, and seek additional resources or support as required. This proactive approach cultivates a more engaged learning attitude, which is vital for the development of independent and competent medical professionals.

Academic and pastoral support

Progress testing significantly strengthens academic and pastoral support by encouraging a collaborative learning atmosphere. This is especially true as students across different years take similar tests, creating a shared experience that encourages senior students to share knowledge and guidance with juniors. This peer-to-peer teaching and mentorship, whether through organised study groups, mentorship programs, or informal sessions, fosters a supportive and inclusive educational community [27]. 

As discussed, progress tests are essential for program staff to consistently monitor student performance. They provide detailed data on each student's academic progress, helping educators identify patterns and scope for improvement. When there's a significant decline in a student's performance, it triggers the activation of pastoral support. This may include support mechanisms like one-on-one counselling, specialised academic assistance, or mental health resources, tailored to the individual's needs. This proactive approach ensures students receive the necessary support to address their challenges effectively, improving their overall well-being and academic success [28].

Quality assurance

Quality assurance in progress testing, particularly in medical education, is comprehensive and vital. Preliminary studies suggest that tests consisting of over 120 questions and conducted multiple times a year ensure thorough curriculum coverage and enhance the reliability and validity of the assessment [17]. The creation of a diverse and robust question bank is key to effectively evaluating students' medical knowledge and skills. This bank should include various types of questions, align with curriculum objectives, and be updated regularly to stay current with medical practice [17]. This helps minimise recall bias and maintains the relevance and challenge of the questions.

Collaboration between medical schools is beneficial in addressing quality assurance challenges. Schools can either share question banks or form a collaborative board to create and maintain a high-grade question pool, as demonstrated by the Medical Schools Council's Assessment Alliance [29]. This collective effort leverages a wider range of expertise in question creation, thus enhancing testing standards. It also optimises standardisation, allowing for more accurate comparisons between students from different medical schools when necessary.

In the UK, the introduction of the UK Medical Licensing Assessment (UKMLA) from 2025 marks a significant advancement in medical student assessment. The Applied Knowledge Test component of the UKMLA will effectively build upon progress testing as it will be developed through collaboration among the country's medical schools. This will allow each medical school to bring their expertise and experiences of progress testing to implementing this new assessment method [30]. While a detailed analysis of the UKMLA's implementation is outside this review's scope, its introduction indicates a move towards more standardised, quality-assured assessments in medical education [31]. 

## Conclusions

Progress testing in UK medical education presents a transformative approach, aligning more closely with the dynamic and comprehensive needs of medical training in the 21st century. By adopting a constructivist framework, progress testing offers a consistent and contextual method of assessment, which gauges not only students' knowledge but also their capability to apply this knowledge in clinical scenarios. This approach fosters a deeper understanding and retention of medical concepts, thereby equipping students more effectively for the complexities of their future roles as doctors. The continuous nature of progress testing, combined with its comprehensive coverage of the medical curriculum, ensures that learning is ongoing and integrated, rather than fragmented and exam focused. This shift from traditional examination methods to a more holistic and continuous assessment strategy not only enhances the quality of medical education but also nurtures the development of well-rounded, competent medical professionals.

Furthermore, progress testing serves as a catalyst for curricular innovation, driving medical schools to adapt their teaching methods and content to this continuous assessment model. The feedback and data generated from these tests provide invaluable insights for both students and educators, enabling a more targeted and effective educational experience. This model also aligns well with the regulatory standards set by bodies like the GMC, ensuring that medical education in the UK remains at the forefront of global medical training. As the medical landscape evolves, so must the methods of educating future doctors. Progress testing, with its emphasis on continuous learning and application, represents a significant step forward in adapting medical education to meet the demands of contemporary healthcare.
